# Prize Essay Matters

**Published:** 1867-06

**Authors:** 


PRIZE ESSAY MATTERS.

The following resolution, offered yesterday, was taken up:

*Resolved*, That hereafter the Committee on Prize Essays be requested to withhold the offer of a prize of $100 each, and that the successful essayists receive in lieu thereof a certificate signed by the President and Secretary, and one hundred copies of the essay, at the expense of the Association.

This resolution was suggested by the failure of the receipts of the Association to pay printing expenses.

Dr. Davis made a statement to the effect that if the different sections would conform to the ordinances of the Association, as to proper matter to be referred for publication, the expenses of printing would be greatly reduced, and the prizes could be paid.

Dr. Robbins called attention to an amendment that had been proposed two years ago, requiring all members, whether present or absent, to pay an annual fee of $5. By the adoption of this amendment the Association would have ample funds.

The subject was laid on the table until the amendment could be laid before the Association in proper shape.

